# Stroke-Like Lesion in an m.3243A>G Carrier Presenting as Hyperperfusion and Hypometabolism

**DOI:** 10.7759/cureus.15487

**Published:** 2021-06-07

**Authors:** Josef Finsterer, Martina Kudlacek, Siroos Mirzaei

**Affiliations:** 1 Neurology, Krankenanstalt Rudolfstiftung, Vienna, AUT; 2 Nuclear Medicine, Wilhelminenspital, Vienna, AUT

**Keywords:** mtdna, m.3243a>g, melas, multisystem, stroke-like epsuode

## Abstract

Carriers of the m.3243A>G variant typically manifest with stroke-like episodes (SLEs), of which the morphological correlate on imaging is the stroke-like lesion (SLL). The pathophysiology of SLLs is poorly understood but acute and chronic stages are delineated. Here we present the case of an m.3243A>G carrier who presented with hypometabolism during his second SLL.

The patient was a 56-year-old male who was diagnosed with MELAS (mitochondrial encephalopathy, lactic acidosis, and stroke-like episodes) at the age of 50 upon a third SLE, muscle biopsy, and the detection of the m.3243A>G variant in the muscle. A fluorodeoxyglucose-positron emission tomography (FDG-PET) during the second SLE revealed hypometabolism in the occipital lobes bilaterally. The patient was misdiagnosed for years and was repeatedly exposed to mitochondrion-toxic drugs (metformin, steroids, valproic acid, oxcarbazepine, zolpidem). The previous data and the present findings indicate that the hypometabolism on FDG-PET together with reduced oxygen-extraction fraction (OEF) on OEF-MRI and hyperperfusion on perfusion-weighted imaging (PWI) characterise best the acute stage of an SLL.

In conclusion, an acute SLE in m.3243A>G carriers typically manifests with a mismatch between hyperperfusion on PWI or single-photon emission computed tomography (SPECT) and hypometabolism on FDG-PET and hypointensity on OEF-MRI. Since SLEs are not vascular events, they should be managed by a multispecialist approach and not by general or stroke neurologists.

## Introduction

The variant m.3243A>G manifests with syndromic or non-syndromic phenotypes [1]. Among the syndromic phenotypes, mitochondrial encephalopathy, lactic acidosis, and stroke-like episodes (MELAS) syndrome is the most prevalent [2]. The most typical phenotypic manifestation of MELAS is the stroke-like episode (SLE), of which stroke-like lesion (SLL) is the morphological equivalent on imaging [3]. Though SLLs are poorly defined [1,2,3], an acute and a chronic stage can be delineated. Here we present an m.3243A>G carrier who presented with hypometabolism during the acute stage of an SLE.

## Case presentation

The patient is a 56-year-old Caucasian male (height: 173 cm, weight: 53 kg) with a history of visual impairment on the right eye since childhood, bilateral hypoacusis, and cholecystectomy. At age 42 years, he presumably experienced the first SLL in the right parieto-occipital area manifesting with a transient flickering of the left eye, quadrantanopia to the lower left, left-sided ataxia, depressive mood, multimodal memory deficits, and a holocrane headache. Echocardiography revealed concentric thickening of the left ventricle in the absence of arterial hypertension. Mild hyperCKemia and hyperlipidemia were noted for the first time.

**Figure 1:**
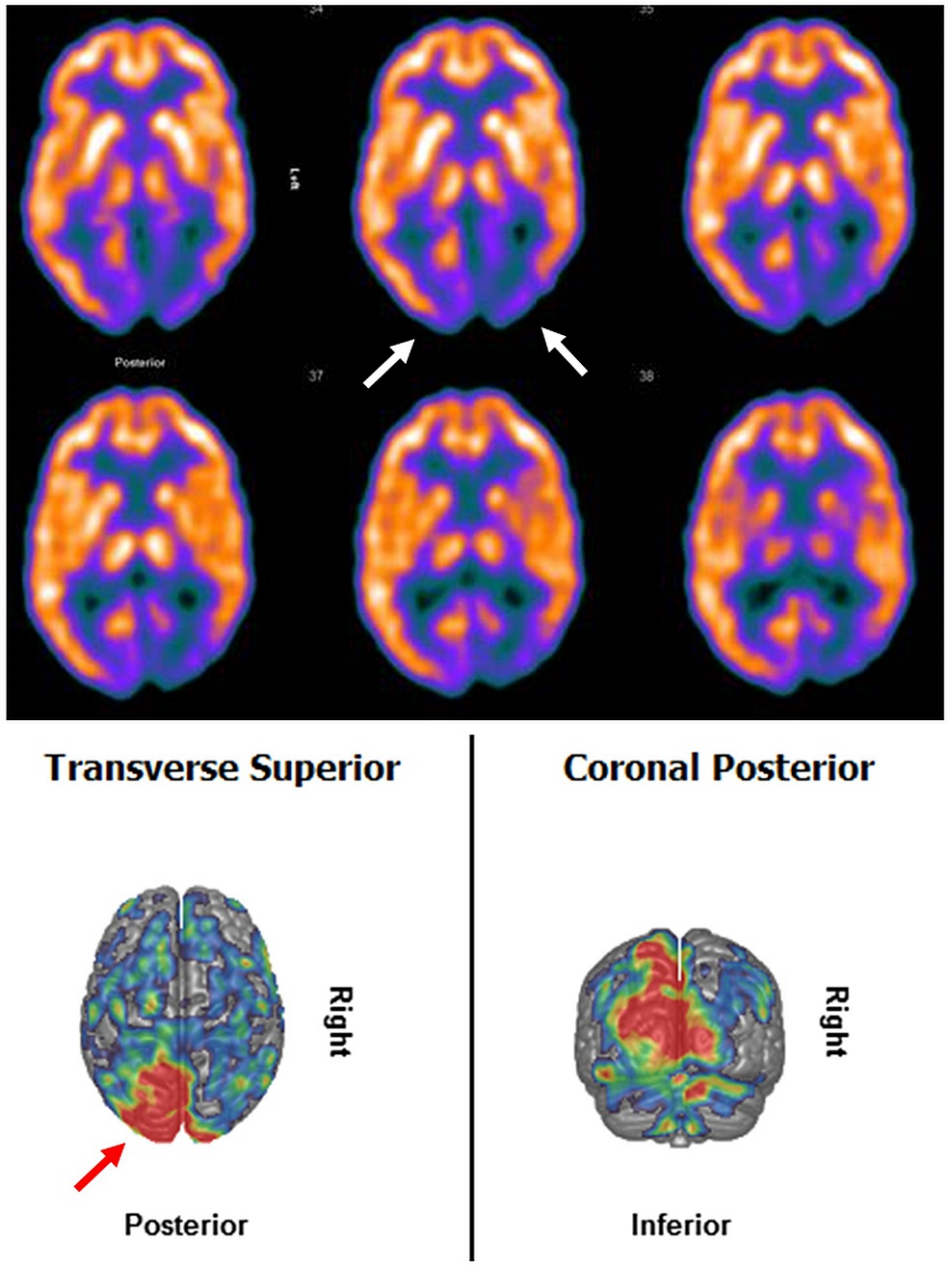
Occipital hypometabolism on 18F-FDG PET (301 MBq 18F-FDG, Siemens Exact, Knoxville, USA) with left-sided predominance during the second SLE at age 49 years. Upper panel: trans-axial slices with violet areas indicating hypometabolic occipital cortex. Lower panel: 3D cortical surface compared to an age-matched normal collective, red areas indicating significant hypometabolism in the cerebral cortex.

At age 49 years, the patient was admitted for a presumed second SLE manifesting with recurrent, focal myoclonic seizures, right-sided hemiparesis, gait disturbance, memory deficits, and dysarthria. Cerebral MRI showed a T2-hyperintense, cortical, and subcortical lesion in the left parieto-occipital area. Cerebrospinal fluid (CSF) investigations revealed elevated protein and lactate exclusively. Immune-encephalitis was suspected. To exclude paraneoplastic syndrome, a whole-body 18F-FDG-PET was performed. Interestingly, 18FDG-PET revealed hypometabolism in the left>right occipital lobes (Figure 1). The PQ-interval was shortened. Neuropsychological testing revealed a dysexecutive syndrome. Corticosteroids and anti-seizure drugs (ASDs) (levetiracetam, lamotrigine, oxcarbazepine, and valproic acid) were started. Since then, lactate was recurrently elevated in the serum and CSF. Prediabetes under steroids developed. Protein-S deficiency was additionally found.

At age 50 years, the patient experienced a third SLE in the right parieto-occipital area, manifesting with weakness of the left lower leg and seizures. This time, a mitochondrial disorder (MID) was suspected, being confirmed by a muscle biopsy. Genetic workup revealed the MT-TL1 variant m.3243A>G in muscle (heteroplasmy 70%). Since the third SLE, he experienced gait disturbance, easy fatigability, progressive memory disturbance, difficulties in finding words, and recurrent panic attacks, triggered by fear from experiencing a seizure.

At age 56 years, the patient experienced a fourth SLE, manifesting as vertigo, confusional state, speech disturbance, and difficulties in finding words, starting two days prior to admission, in addition to ataxic gait and impaired concentration.

The family history was positive for diabetes (mother), valve replacement (mother), myocardial infarction (mother), and carcinoma (father, mother, sister). His medication included bisoprolol, clopidogrel, metformin, pantoprazole, levetiracetam, pregabalin, and ciprasidone.

**Figure 2:**
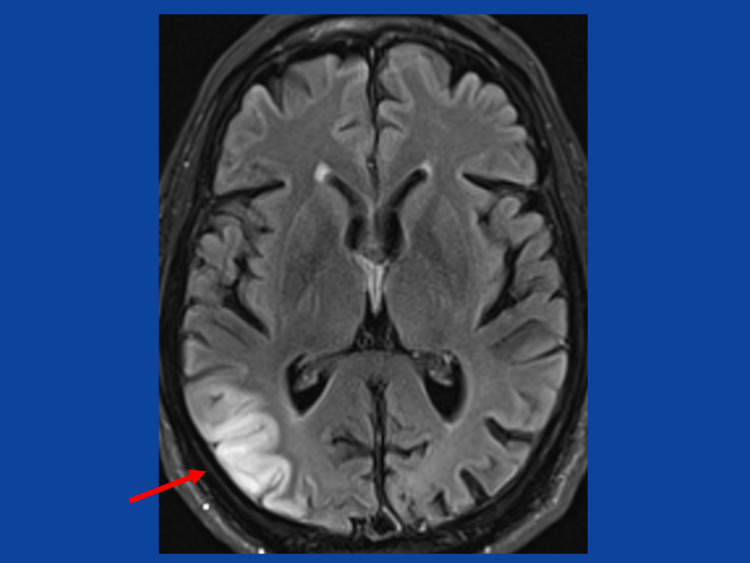
T2-weighted image showing a stroke-like lesion in a right parieto-occipital distribution

Clinical neurologic exam revealed a weakness for head anteflexion (M5-), cerebellar speech, severe visual impairment bilaterally with only light/dark discrimination, severe hypoacusis, mild ptosis bilaterally, diffuse wasting of the upper limbs, bradydiadochokinesia bilaterally, wasting of the thighs, marked ataxia on the lower limbs, and reduced Achilles tendon reflexes. There was an ataxic stance, but he could walk with a walker. Blood chemical investigations revealed lactic acidosis, hyperuricemia, folic acid deficiency, and hyper-triglyceridemia. MRI of the brain revealed an SLL in the right parieto-occipital area (Figure 2) and an asymptomatic T2-hyperintense cystic lesion in the right tongue root. Abdominal ultrasound revealed steatosis hepatic and benign prostate hypertrophy. Diabetes was excluded and metformin discontinued. Folic acid and ezetimibe were added.

## Discussion

The presented patient is interesting for multisystem MID due to the variant m.3243A>G, phenotypically manifesting in the brain in recurrent SLEs, ataxia, epilepsy, cognitive impairment, psychiatric disease, eyes (visual impairment), ears (hypoacusis), endocrine system (hyperlipidemia, pre-diabetes), heart (hypertrophic cardiomyopathy, short PQ, incomplete right bundle branch block), muscle (myopathy), and the peripheral nerves (neuropathy). Protein-S deficiency was regarded rather as second trouble than causally related to the mitochondrial DNA (mtDNA) variant. Polyneuropathy has been previously described as a manifestation of MELAS [4].

The most interesting feature of the index patient was occipital hypometabolism on FDG-PET during the second SLL at age 49 years. Hypometabolism within SLLs has been previously reported and can be explained by the reduced ability of the mitochondria to utilise glucose [5,6]. Hypometabolism could be also due to the replacement of neurons by glial cells [7]. However, since the patient presented with a typical SLE, hypometabolism is rather attributable to the focal metabolic breakdown than scarring. A further argument against a scar is that the left-sided lesion was transient and not seen on imaging during the first, third, and fourth SLEs. Hyperperfusion during the acute stage of an SLL, as has been previously reported [8], can be documented on PWI or SPECT, and can be interpreted as a compensatory mechanism for the focal, metabolic derangement. In accordance with hypometabolism, oxygen extraction is reduced within the SLL, particularly at the onset of an SLL.

The patient is a typical example a year-long misdiagnosis and unnecessary investigations and therapies. Early diagnosis of MELAS is warranted to prevent patients from being exposed to mitochondrion-toxic drugs, such as metformin, steroids, valproic acid, oxcarbazepine, zolpidem, and from undergoing repeated unfocused, costly investigations. Why the patient received metformin despite having been diagnosed with MELAS remains unknown. It is well known that metformin may worsen MELAS [2].

Whether the tongue-root cyst was part of the phenotype remains speculative. An argument in favour of a causal relation is that cysts in MIDs have been reported in the brain, liver, kidneys, pancreas, thyroid gland, and bones. In the brain, cyst-like lesions have been reported in the white matter of MELAS patients. Cyst formation can be attributed to impaired cell adhesion, which may require adequate adenosine triphosphate (ATP) supply for appropriate functioning.

Limitations of the study are that the index patient had not undergone perfusion studies or OEF-MRI during any of the four SLLs and that the SLLs were never treated with L-arginine or antioxidants.

## Conclusions

In conclusion, this case shows that acute SLLs present with a mismatch between hypometabolism on FDG-PET and hyperperfusion on PWI/SPECT. Patients with an acute SLE should undergo perfusion studies and FDG-PET to clearly delineate an SLL from ischemic, vascular lesions. The treatment of SLEs is at variance from that of an ischemic stroke.
